# Rurality and social innovation processes and outcomes: A realist evaluation of rural social enterprise activities

**DOI:** 10.1016/j.jrurstud.2021.04.006

**Published:** 2023-04

**Authors:** Artur Steiner, Francesca Calò, Mark Shucksmith

**Affiliations:** a Yunus Centre for Social Business and Health, Glasgow Caledonian University, M201 George Moore Building, Cowcaddens Road, Glasgow, G4 0BA, UK; b Public Leadership and Social Enterprise, Open University, Walton Hall, Milton Keynes, MK7 6AA, UK; c Newcastle University, Newcastle Upon Tyne, NE1 7RU, UK

**Keywords:** Rural social innovation, Realist evaluation, Social enterprise, Neoliberalism

## Abstract

Although increasingly prominent in research, policy and practice, little is known about social innovation in a rural context. To address this knowledge gap, our paper explores *how rurality might affect the social innovation process*. Drawing on 68 interviews carried out with beneficiaries, service providers and external stakeholders of a rural social enterprise initiative in Scotland, the paper adopts a realist evaluation theory (Pawson and Tilley, 1997) approach combined with Calò et al.’s (2019) social innovation analytical framework to identify Context-Mechanism-Outcome configurations for rural social innovation. The findings highlight that specific characteristics of rural places can act as stimuli of social innovation. Positive outcomes of a social innovation can potentially be rooted in rural peculiarity and its problematic context. Push factors, born out of necessity, lead to *reactive social innovation* and pull factors, derived through harnessing perceived opportunities in the environment, lead to *proactive social innovation*. Importantly, push factors do not undermine the establishment of social innovation – indeed, they can actually promote social innovation and strengthen its validity. The paper also shows that outcomes of the social innovation process might not be specific to rural areas. Instead, the pathway to the desired outcomes is conditioned by rural factors, shaping the contexts and mechanisms of rural social innovation. As different rural locations might have different resources to address local challenges, social innovation processes vary from one case to another, although the challenges being addressed might be similar. As such, rural social innovation policies should not be ‘over prescribed’. Context creates both challenges and solutions and influences the type and form of mechanisms used to achieve a desirable social innovation outcome.

## Highlights

• Rural places can act as stimuli of social innovation.
• Positive outcomes of a social innovation are frequently rooted in rural peculiarity.
• Push factors can promote rural social innovation strengthening its validity.
• Rural social innovation policies should not be ‘over prescribed’.
• Rural context influences the mechanisms used to achieve social innovation outcomes.

## 1 Introduction

Social innovation is a concept whose popularity has increased in recent years, gauged by the number of papers and research centres that have quite suddenly emerged in this prominent but still growing area of interest. Indeed, the term is widely used by policymakers who encourage social innovation practice (European Commission, 2020; Sinclair and Baglioni, 2014). Although popular as a rapidly growing field of academic enquiry (Pol and Ville, 2009; Vanderhoven et al., 2020; Ziegler, 2017), social innovation in a rural context remains mostly unexplored, with very little academic research dedicated to the theme (see, for example, Castro-Arce and Vanclay, 2020; Richter, 2019). Little is known about the impact of rurality on social innovation or how rural contexts affect the processes and outcomes of social innovation. This knowledge gap is surprising considering that, arguably, rural locations have the potential to offer fertile ground for social innovation to thrive. For example, the high level of social capital, cohesion, embeddedness and mutual knowledge among members of rural communities could act as stimuli to social innovation (Steiner et al., 2019), helping to address socio-economic challenges and geographical disadvantages faced by rural residents (Anderson and Lent, 2019; Best and Myers, 2019). The aim of this paper, therefore, is to explore *how rurality might affect the social innovation process*. To do so, we use primary data from a social enterprise study conducted in rural Scotland and, utilising both a realist evaluation theory lens (Pawson and Tilley, 1997; Wong et al., 2017) and Calò et al.’s (2019) social innovation analytical framework, identify rural ‘CMO configurations’ (Context-Mechanism-Outcome). In doing so, we generate new knowledge in the field of rural social innovation.

The paper begins by explaining the importance of the rural context, establishing a description of social innovation, and reviewing the literature that focuses on the potential of rural social enterprises to support socially innovative activities. It continues with an outline of realist evaluation theory and the research methods adopted in our study. The findings are then described and presented in terms of context-mechanism-outcome configurations, which are used to inform the development of a programme theory. After discussion of the findings, we explore how the characteristics of rurality might impact on social innovation processes and with what implications for policymakers and researchers. This knowledge serves as an important basis for future research exploring the means by which rurality could affect the outcomes achieved by rural social innovation initiatives.

## 2 Rurality and social innovation

### 2.1 Challenging but not always bad – features of rurality

In the late 20th and early 21st centuries, aspects of technological innovation, changing socio-economic and political contexts, as well as globalisation have transformed the way rural localities operate, influencing the lives of local residents (Steiner and Atterton, 2015). Practitioners and academics alike have discussed both the positive and negative consequences of these recent changes and their impact on rural community resilience (Imperiale and Vanclay, 2016; Markantoni et al., 2018; Shucksmith, 2018).

Rural communities are frequently characterised by a high level of social cohesion associated with a commitment to self-help and active civic participation (Farmer et al., 2008). Embeddedness, strong mutual knowledge and a sense of community can lead to high levels of trust among rural residents (Granovetter, 1985; Jack and Anderson, 2002) who are willing to collaborate and collectively address challenges (Zografos, 2007). Reciprocity, collective activity and social capital translate into dense social networks (Richter 2017, 2019) with rural citizens being more socially orientated in their entrepreneurship than those living in urban locations (Muñoz et al., 2015; Williams, 2007).

Nonetheless, despite these positive features that could promote social innovation, the geographical nature of rurality frequently leads to deprivation or disadvantage (Shucksmith et al., 2021a). Rural places tend to share a set of challenges including outmigration of young people and concentrations of older people, limited employment opportunities, and difficulties attracting specialist workers (Christmann, 2017; O'Shaughnessy et al., 2011). Small and widely dispersed populations mean that commercial enterprises cannot take advantage of economies of scale, limiting their profitability and willingness to invest in rural locations. Simultaneously, high per capita public service provision costs lead to the closure of economically unviable services, especially in the context of neoliberalisation and austerity. For instance, we see evidence of healthcare organisations being moved to larger regional centres, leaving rural residents with limited (or no) primary healthcare services (Farmer and Nimegeer, 2014). In many rural places, village halls, churches, local pubs, schools, libraries, shops, post offices, transport as well as emergency services have been withdrawn. These may both be seen as part of the ‘roll-back’ phase of neoliberalisation characterised by cutbacks in public spending, privatisation, deregulation and the dismantling of the institutions of Keynesian welfarism (Peck and Tickell, 2002). Such economic pressures create a barrier to local development, with many services and goods being inaccessible to those living in rural places. Diminishing public resources limit opportunities for social interaction and erode the strong social ties once associated with rural communities. Many agricultural and fishing villages as well as rural market towns have become commuter towns, places to retire, or tourist destinations (Skerratt, 2012). It could be argued, therefore, that social connections created through a sense of shared identity no longer provide the social capital that previously bound these communities together, with some rural residents experiencing social exclusion (Shucksmith, 2004).

At a global level, factors such as economic downturn, health pandemics, public spending cuts, ageing populations and climate change also have a direct negative impact on rural localities, accelerating existing challenges. Meanwhile, the ‘roll-out’ phase of neoliberalisation introduces new modes of governance and re-regulation, encouraging voluntary and community organisations to act competitively rather than collaboratively, to earn income through tenders and contracts, to pursue targets and deliverables perhaps tangential to their purpose, and to adopt the modes and practices of business to pursue the agendas of the neoliberalised state (Shucksmith et al., 2021b). See Although this asymmetrical power relationship can limit the potential for localised or ‘bottom-up’ action (Peck and Tickell, 2002), there are also opportunities emerging for multiple, local (perhaps rural) forms of development, rooted in local cultures, values and movements (Peck et al., 2010).

No matter whether rurality is presented in a positive or less positive way, one thing is clear – rural context matters and creates a specific environment that has an impact on local activities (Steinerowski and Steinerowska-Streb, 2012). Considering this, we question how the context of rurality affects the processes and outcomes of social innovation.

### 2.2 Social innovation – what do we know and what is unknown?

In many countries around the globe, policymakers attempt to foster social innovation in order to increase the self-reliance and sustainability of their communities (Dodgson et al., 2011; European Commission, 2020; Goldenberg et al., 2009). Despite its popularity, the concept of social innovation remains fuzzy, lacking one universally agreed definition (Ayob et al., 2016). For example, social innovation could be regarded as the development of new ideas/products/services, the improvement of actions/processes, the use of new, creative and novel practices or even increased democratic participation that aims to build a more equitable, fair, efficient and sustainable society (Castro-Arce and Vanclay, 2020). This all-encompassing description of social innovation has led to some commentators referring to the concept as a buzz word and passing fad that is ‘too vague to be usefully applied to academic scholarship’ (Pol and Ville, 2009, p.878). Nonetheless, as a type of innovation that focuses on the creation, renewal or transformation of social relations to facilitate new ways of working together and achieve societal goals (Moulaert et al., 2013) and that addresses community needs through community empowerment (Bock, 2012, 2016), social innovation attracts growing academic interest.

While acknowledging the wealth of definitions of social innovation, in this paper we take a pragmatic approach and consider social innovation to be a normative concept that involves collaborative actions and participatory processes that, through satisfying social needs and achieving common desires and aspirations, help to improve society (Moulaert et al., 2017). Rather than researching the broad transformative impact of social innovation (Avelino et al., 2019; Westley et al., 2017), we explore social innovation at a micro-rural-scale. The latter is important, as once novel, local level initiatives are interwoven across geographical scales and political levels, they can help to achieve systemic change (Parés et al., 2017).

Particularly relevant in the rural context – and something that is widely described in the rural community resilience literature (see, for example, Davoudi, 2012; Imperiale and Vanclay, 2016) - is the perception of social innovation as a way of adapting to change and bouncing forward (Steiner and Markantoni, 2014). Hence, social innovation can be *reactive*, tackling current and pressing issues, as well as *proactive*, searching for new and more sustainable ways of working, as well as *transformative* (Davoudi, 2012). In both cases, essential in sparking social innovation is a collaborative space that facilitates the engagement of actors at different political levels, geographical scales and across industrial sectors (Neumeier, 2017). Triggered by local interests, bottom-linked governance mechanisms, and by time and place-specific contexts, social innovation can foster regional development (Castro-Arce and Vanclay, 2020; Christmann, 2014). As such, geographical features of rurality highlighted in the literature are relevant to our rural social innovation study.

One difficult issue is whether social innovation is complicit in neoliberalisation, facilitating the roll-back of the state by mitigating and masking its impacts, or a transformational source of alternatives and resistance to neoliberalisation. Cheshire (2006), for example, warns of governments promoting narratives of endogenous community self-help as a means of reducing public expenditure and transferring responsibility for provision of services and infrastructure to rural people themselves, aided by a belief that self-help was a natural state for rural communities. Academics who promote self-help uncritically, she argued, risk becoming ‘cheerleaders for neoliberalism’.

### 2.3 Rural social enterprise and social innovation

Social enterprises can be perceived as drivers of social innovation initiatives. Internationally, social enterprises are increasingly promoted as a potential response to individual and place-based disadvantage and as a way to increase community capacity and address public health and wellbeing problems (Calò et al., 2018; Kelly et al., 2019). This is premised on the proposed benefits arising from encouraging citizens to take responsibility for providing required goods and services in a flexible manner and through the sustainable use of local resources (Nicholls, 2010; Teasdale, 2012). Indeed, there is evidence indicating that social enterprises stimulate citizen and community participation, thus filling service gaps left by the state and commercial businesses and bringing about positive local transformations (Henderson et al., 2020). These characteristics of social enterprises and their potential impact on health and wellbeing are relevant to our study, though we focus our attention on rurality and social innovation.

Recently, the *Journal of Rural Studies* published a Special Issue dedicated to rural social enterprise (Steiner et al., 2019). This specific paper collection shows that social enterprises could respond to some of the challenges rural communities face and introduce changes needed to become more resilient (Apostolopoulos et al., 2019; Barraket et al., 2019; de Haan et al., 2019; Kelly et al., 2019; Morrison and Ramsey, 2019; Steiner and Teasdale, 2019). Interestingly, although social enterprises could be perceived as a vehicle that facilitates the process of social innovation, only a few contributors to the special issue directly referred to social innovation (Anderson and Lent, 2019; Best and Myers, 2019; Richter, 2019). For example, Richter (2019) suggests that social enterprises are more capable of fostering social innovation if they are socially embedded in the region and if they systematically connect remote rural communities with groups, organisations, and networks in other places, fields, and spatial scales. As such, fostered through rural social enterprises, networking is essential in facilitating social innovation. Being widely embedded in rural regions, rural social enterprises can harness the opportunity to identify social needs, then develop and find local support for the implementation of innovative solutions (Richter, 2017, 2019).

Indeed, as businesses with primarily social objectives and an organisational format that uses trading to tackle social, economic and environmental challenges (Teasdale, 2012), social enterprises seem to offer novel practices, with local actors identifying new or more efficient ways of working that foster social change and bring positive social impacts (O'Shaughnessy & O'Hara, 2016). As innovation is related to a specific setting and based on the recombination of existing elements and the transfer of ideas and solutions to or from other contexts, rural social enterprises can innovate through ‘re-contextualisation’ of ideas that already exist in other contexts (Richter, 2019). Considering the importance of the local context and the embeddedness of local actors, Richter (2019, p.185) stresses that social innovation introduced by rural social enterprises is not ‘simply copied but adjusted according to the preconditions in the new context in order to meet specific needs, spur acceptance, safeguard the resources necessary for the implementation, and optimise the outcome.’ As such, to innovate in a local area, one needs to be aware of the local needs, identify an innovative idea and adapt it to local settings.

**Fig. 1:**
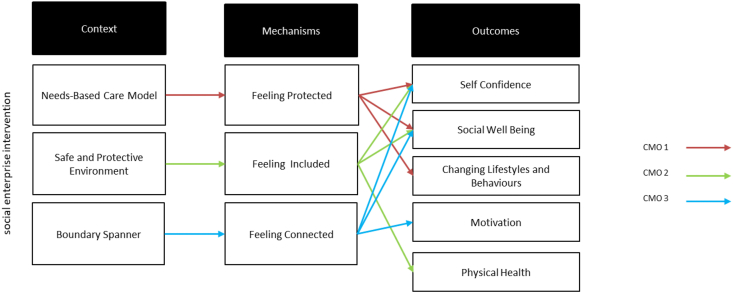
Context, mechanisms and outcomes of a social innovation initiative (Calò et al., 2019).

However, while a social enterprise model may introduce social innovation and contribute to local development, the capacity of rural social enterprises to achieve transformational change is questioned (Cieslik, 2016). In reality, aspects of rural social enterprise and *rural social innovation* are largely unexplored, with limited studies focusing on the rural context (Anderson and Lent, 2019; Richter, 2018, 2019). Considering this background, we explore *how rurality might affect the social innovation process*. In particular, using realist evaluation theory we aim to identify how the rural *Context* in which social innovation develops affects its *Mechanisms* (e.g. processes) and *Outcomes* (e.g. what is achieved). Our paper builds upon and expands Calò et al.’s (2019) analytical framework (Fig. 1) adding a new specific focus on rurality as one of the specific characteristics of the context in which the social innovation initiative took place.

## 3 Methodology and methods

Realism has been seen as a means of bridging two opposing paradigms - the positivist and the interpretivist - and was developed in response to the perceived limitations of each (Ackroyd and Fleetwood, 2000; Blackwood et al., 2010; Creswell and Clark, 2010). Realism is thought to draw upon the merits of both objectivist and subjectivist approaches to reflect the complex interplay between individual, organisational, and programme-related factors (Clark et al., 2007). Consistent with the positivist approach, realism accepts the existence of one stable reality but it does not espouse the objective acceptance of all research findings (McKendrick, 1999; Modell, 2009).

Realists argue that causal connections are established via ‘context, mechanism, outcome configurations’ (Pawson, 2006, p. 25), with the three components working together to explain a causal effect in a stable reality. Realist evaluations are based on the identification of outcome patterns, generative mechanisms and contextual conditions, which help to assess not only ‘what works’, but also for whom, and in what circumstances (Blackwood et al., 2010; Pawson, 2006; Pawson and Manzano-Santaella, 2012; Pawson and Tilley, 1997). This approach focuses on building and refining programme theories concerning complex casual mechanisms and exploring how these mechanisms interact with contextual and individual characteristics (Fletcher et al., 2016). The task of a realist evaluation is to explore the ‘black box’ of programmes (Salter and Kothari, 2014), identify how an intervention is supposed to work and whether the underpinning theories concerning mechanisms, context and outcomes are plausible.

So far, only a few papers have used realist evaluation to explore social innovation initiatives (Milley et al., 2018). Some have investigated the generative mechanisms involved in achieving physical health, mental health and social determinant outcomes by interviewing managers of various organisations (Roy et al., 2014). One paper explored the contribution of social enterprise to health and social care (Calò et al., 2019). Very few studies have used realist evaluation to explore programmes undertaken in rural areas (e.g. Adams et al., 2016; Zhao et al., 2017) and none of these has focused on social innovation initiatives or social enterprises. In this paper, a realist approach is used as it helps to explore the role of rurality in affecting the social innovation process.

### 3.1 Research setting

The focus of our paper is on a social innovation initiative - Active Life (name has been changed to preserve confidentiality) - that has been operating in a rural community in Scotland for more than six years. As such, we explore the micro-rural-scale (Parés et al., 2017) of the social innovation process. The main aim of Active Life is to increase the physical activity levels of people with chronic health conditions. The initiative, run as a social enterprise, was created through a partnership between the local medical centre, the physiotherapy and dietetics departments of the local hospital, and another well-established community-based organisation operating in the area. The service represents a form of social innovation (Moulaert et al., 2017), building collaboration between actors from the public sector (including a GP practice and a local hospital) and a social enterprise run by the local community in order to address health challenges faced by rural residents.

The area where the social innovation takes place is characterised by a proportionately high number of people dealing with at least one chronic condition. Active Life has therefore been developed as an addition to existing health care services, reinforcing the links between local NHS workers and the community. The services are run as a GP referral scheme, providing classes and one-to-one services to local people with chronic conditions. Starting with a consultation with a fitness instructor, a physical activity programme is then tailored towards an individual's needs. The initiative was purposively selected as our case study as it enabled partnership between a social enterprise and the public sector and was created to combat specific health challenges (i.e. the rate of chronic conditions in the area).

### 3.2 Data collection

Our realist evaluation was based on 42 in-depth semi-structured interviews with relevant stakeholders including project managers (n = 4), health professionals (n = 6), beneficiaries (n = 22), project leaders (n = 4), managers of the main partner organisations (n = 4) and grant-makers (n = 2).

This group of stakeholders was selected to capture different points of view (Brandon, 1998). Thirty-eight of the interviews were recorded and transcribed ‘intelligent verbatim’, while the remaining three interviews were recorded via extensive field notes. Interviewees were asked between 3 and 9 open-ended questions, depending on their stakeholder group. Consistent with the realist approach, the interview guide was divided into the three domains of inquiry: (i) identifying generative mechanisms and how they trigger the achievement of outcomes; (ii) understanding how contextual variables influence the outcomes of the intervention; and (iii) exploring the outcome patterns of the intervention. In addition, we conducted one week of observations, following one of the entire project leaders in all her activities (from the first meeting with beneficiaries to physical activity courses and community classes) to better understand the activities in which beneficiaries usually take part (Bryman, 2012). Details of our interviews and observations is presented in Table 1.

**Table 1:** Data collection details.

Data	Participants	Number of interviews/observation
Interviews	Project Managers	4 Interviews
	Health Professionals	6 Interviews
	Project Leaders	4 Interviews
	Beneficiaries	22 Interviews
	Main Partner organisation	4 Interviews
	Grant Makers	2 Interviews
Observation	Project Managers/Health Professionals and Beneficiaries	1 week of observations of all the activities conducted by the project managers

Confidentiality of all study participants was ensured and, as such, we do not reveal the real names of interviewees. In addition, all information that could compromise the anonymity of study participants has been removed. Ethical approval was requested and obtained from the NHS Highland Research & Development Committee and the [Name of the Department of the University blinded for reviewers].

### 3.3 Data analysis

The interviews were transcribed by the lead researcher and, after ensuring that the transcripts were an accurate record of each interview, the data were imported into the computer-assisted qualitative data analysis software QSR NVivo for three cycles of analysis. For the first cycle, causation coding driven by the domains of inquiry was used to identify context, mechanisms and outcomes (Saldaña, 2021). During the second cycle, pattern coding was employed to group concepts together (Miles and Huberman, 1994) and establish what works, for whom, and in what circumstances.^1^ During the third cycle of analysis, used to inform this paper, data were reanalysed to consider the importance of rurality in affecting the mechanisms and outcomes of the social innovation initiative. This third cycle of coding employed ‘linked coding’ which involves ‘sticking very closely to the descriptive accounts of the interviewees’ (Jackson and Kolla, 2012, p. 342), to generate CMO configurations from the narrative accounts of those interviewed.

## 4 Findings

The findings have been grouped into three CMO configurations. The first grouping relates to the theme of *developing new social innovation initiatives*, the second relates to *the increasing embeddedness and social cohesion inside the initiative*, and the third relates to *rising reciprocity, collective activity and social capital in the community*. Each is explored in turn.

### 4.1 Developing new social innovation initiatives

**CMO 1:** The rural location where the initiative took place was characterised by stakeholders as lacking exercise opportunities for older people (context). The lack of relevant services triggered community members to develop a new intervention to address challenges that older people were facing (mechanism). Consequently, funders and managers of the social innovation initiative developed “a needs-based care model” as a solution to addressing the needs of local elderly people with chronic conditions (outcomes).

**Context:** All stakeholders and beneficiaries recognised that the rural location where the social innovation initiative took place did not have specific services supporting the physical activity of older people with chronic conditions. Two local leisure centres were perceived as ‘*places for fit people*’, not easily accessible to those who struggled with their age and health: *“Elderly people involved in the leisure centre did not feel that they have the* support *… [going there] did not make them feel good. The program did not really match … their symptoms … it was not really improving all the symptoms … it was actually making them feel bad ….and that's why they stop going”* [Project Manager]; *“People often think that anybody attending a gym is this lycra dressed fit person who will judge the other people”* [Health Professional 3].

For example, one Active Life beneficiary suggested that before joining the social innovation initiative he struggled to be active, using only his garden to incorporate some physical exercise into his life: *“I have a reasonably large garden, so I work in the garden, I have got a lot of trees and physically chopping my own trees, [the tools are] reasonably heavy to handle … so I obviously walk up and down … but really apart from the odd amount of walking, very little exercise … above a normal life. I would say less than I probably should be doing”* [Beneficiary 1].

The lack of services was particularly evident for people suffering from complex chronic health conditions such as multiple sclerosis. To undertake physical classes and manage the pain and physical fatigue related to their condition, this group of older people was forced to travel to neighbouring cities. Health professionals referred to a 3-h long car journey to access relevant support which, considering experienced health vulnerabilities, was highly challenging.

**Mechanism:** A lack of services combined with knowledge of increasing chronic health conditions among the rural community were the triggers that pushed social innovation leaders to develop a new intervention: *“40% of our local community had one or more chronic medical condition and these conditions could be improved or managed by having an active healthy lifestyle and the realization was that we had assets, the facilities and the resources to do that locally within our community. And it didn't need to be made by the medical professionals, the community could have the answer to this … We discovered this and we felt it was our responsibility to do something about it rather than waiting for others”* [Project Leader].

Leaders of the initiative felt the need to develop new services because they wanted to improve the community where they live, as suggested by one of the health professionals who promoted the initiative: *“We have a group of professionals who value living in [this rural area], they wanted the town to flourish for the population … to meet their potential … they really want the town to become a good place, a healthy place”* [Health Professional 1].

Professionals involved in developing the intervention felt the need to create a service that could help people with long-term conditions and those who were unable to find appropriate services in their communities: *“What we are doing is providing a massive service to those people who have long-term conditions. So, for example, our people who have MS … until [Active Life] started we had very few options … treatment options for people with MS in the area and so … now we probably have a service that is unique in the country … in that we are able to give a sort of assessment and a treatment plan … That treatment plan doesn't involve medicalising them and telling them they have to come to the hospital to do … their treatment … they can actually choose where they have [it]”* [Health Professional 3].

**Outcomes:** “*A needs-based care model*” was identified by social innovation leaders as the best approach to effectively delivering services needed by the local community. Indeed, the initiative focused on and was based on the needs of service users: *“We made this model of instead of the client having to go to the place where they were going to do the physical activity … they met their instructor actually on the GP premises and they were given an option of a home-based programme or a centre-based programme or a group programme, so it was not a purely gym-based focus”* [Health Professional 1].

Promoting “*a needs-based care model*” facilitated alteration of processes and services as soon as new service user needs were identified. For example, the initiative moved from an initial physical activity programme to develop a falls prevention intervention and a counterweight service: *“This community-driven initiative was led by pockets of findings … that's why we have moved to a kind of falls prevention and a counterweight … it is a different strain of what we initially had thought of but actually it is very, very appropriate”* [Health Professional 1]. For each beneficiary, an exclusive programme based upon their needs and limitations was developed: *“They find my limits and they stick around them, they don't push beyond that … and they always give me the option to expand [their programme] if I feel I am able to”* [Beneficiary 2].

According to one social innovation leader, developing a needs-based care model and an innovative way of delivering public services was possible due to the flexibility that a small community-based organisation could offer: *“One of the benefits of a small community-based organisation is that we are independent and can respond quickly to the changes we see … Our experience or my experience of the third sector, is that we have no hierarchy … once we have analysed and come to a conclusion about what we should do, we are then in a position to do it … so we don't have to go to committees and there is a freedom … a freedom to act. What we are able to offer is that flexibility the statutory agencies are not able to offer”* [Project Leader].

### 4.2 Increasing embeddedness and social cohesion inside the initiative

**CMO 2:** The rural location where the initiative took place was identified by the stakeholders as a cohesive town, where retired people and professionals delivering services live and work with a shared desire to improve the community (context). This context encouraged a sense of community, strong mutual knowledge and embeddedness inside the initiative (mechanism), which then led the initiative to be recognised as a safe and protective environment (outcomes).

**Context:** The location where the social innovation initiative took place - a rural town – is inhabited by a large proportion of retired people. Rural residents reported being well-connected with their neighbours as well as service providers. Interviewees considered themselves an integrated part of the local community and acknowledged that people know each other well, they trust their service providers, and often volunteer or work to enhance life in the community: *“In general practice in the small town you get to know patients very well, so you started from a level where you've got a good relationship with these people”* [Health Professional 1].

According to one of the managers of the leisure centre, strong interpersonal connections assist local residents in finding solutions and addressing challenges experienced by their community in an effective way: *“If the problem is in the community, the solution is in the community … and we should not sit back and wait for lots of money to be thrown at it and wait for somebody else to do it … You find if you live here people don't wait for somebody else to do it … get off the bumps and they do it and they make it happen and then it makes a difference because these guys are connected enough … everybody is involved to get everybody's points of view and come with a good end product … They talk with everybody and they take everybody on board”* [Manager of the Leisure Centre].

**Mechanism:** Living in a rural town characterised by residents proactively addressing the needs of their neighbourhoods, encouraged a sense of community, strong mutual knowledge and embeddedness. The opportunity to undertake socially-embedded physical exercise together with friends and neighbours helped to increase participation in the intervention and, through moral support, boost confidence and motivation: *“We thought about it before we came … and we thought it would have been good for encouraging one another … I have him on my heels all the time … so he keeps going”* [Beneficiary 3]; *“yeah … it is a lot of moral* support *… We are going around a lot together with also another friend at the moment … we're always golfing together”* [Beneficiary 4]; *“It is easier to do all these things with your peers because you can compete. I got neighbours in there, and yes, I can compete with them; mentally it is fine, I don't do it in an awful way. It is a motivational push, it is psychological, it is better to compete with your own generation if you are going to compete”* [Beneficiary 1].

In addition, the intervention led to opportunities to meet new people and integrate with segments of the community that they would not meet otherwise. This process was important in forming new connections inside the initiative: *“You come in, and the girls speak to you … you are doing well … I speak to people now … I would normally not speak to … so I am certainly feeling a part of it”* [Beneficiary 3]; *“You meet new people … you get to know new people … you make more friends … you can meet up for coffee any time you want”* [Beneficiary 5].

One of the managers responsible for developing the initiative was praised for creating strong ties with beneficiaries. Interviewees also acknowledged that the manager's passion and commitment was essential to successful delivery of the programme: *“I have no doubt whatsoever, if it was not for the commitment and the enthusiasm of the fitness manager, the whole programme would not have the success that it has”* [Health Professional 2].

Knowing people and being part of a group were seen as an important component of the intervention: *“… they have an away day, so they go on the boat, they go in the hotel that has a spa and the swimming pool … and they have a great day out … They bake cakes and they do all sorts of stuff … There is about 15-20 of them. One of the clients says it was the first time with me being normal again and having confidence … and you think that's only incidental, it is not so important as doing exercise and activity, but it is equally if not more important, it is part of the community … Clients and neighbours, their friends. Everything that I think it is an example of how Active Life and the clients can* support *and help each other”* [Project Leader].

**Outcomes:** The sense of community, strong mutual knowledge and embeddedness generated a protected and non-judgemental space in which beneficiaries felt listened to and safe to participate in physical exercise: *“After you have a heart attack, you get scared to do things, in case something is going to happen. When you go to Active Life, they are with you, they push you … they seem to stretch your ability … I had a few episodes in there where I have not really been very well. We just stop immediately. They take me aside. They look after me”* [Beneficiary 2].

### 4.3 Rising reciprocity, collective activity and social capital in the community

**CMO 3:** The rural location where the intervention took place is characterised by the long-standing influence and vibrant activities of third sector organisations and social enterprises (context). This specific characteristic triggered reciprocity, collective activity and increasing social capital (mechanism), which led to the social innovation initiative being a ‘boundary spanner’ and creating relations within and between different community actors (outcome).

**Context:** The rural location where the social innovation initiative took place was seen as fertile ground for developing third sector community initiatives. These collaborations and activities were supported by a variety of local professionals and residents interested in making a positive change in their rural town: *“Active Life recognises the value of … good partnership … [with] other community groups … You've got youth café, the cinema, the leisure centre, you've got all the youth organisations working together, you got all the health organisations working together, all the third sector health organisations - we know each other, we can pick up the phone, we can speak to them … During the two-year pilot we had 25 different health professionals attending our program and patients coming in … it is evidence that there is willingness to* support *and to help each other”* [Project Leader].

Interestingly, some respondents recognised the uniqueness of the rural location in promoting a fertile environment for developing social innovation initiatives: *“It is fair to say [name of the town] is quite different to many rural areas, it has very established partnership working and there is a lot of social enterprises here”* [Manager of Leisure Centre].

**Mechanism:** A favourable rural setting supported the development of reciprocity, collective activity and social capital in the community. As indicated by one of the founders of the initiative, having previous experience and know-how in running social enterprises in the same town was helpful in identifying and accessing local resources. All the assets of the community were bound together synergically. For example, thanks to close collaboration with the medical centre, health professionals were more motivated to refer beneficiaries to the initiative. Involving the leisure centre provided the physical spaces where the activities were conducted. *“Having the leisure centre, it is fantastic and that helps how we work. Also being a small community and having one medical centre, one GP surgery … is good … It is easy to work with and they are quite motivated to go ahead and so having that relationship is good. And having the three of us, which were previously directors of the leisure centre and one of us being the senior practice nurse of the surgery, all make it easy to work together … This local connection mix helps Active Life to do what it does. There has not been pressure about who takes the credit, as long as the clients, the patients benefit we don't care”* [Project Leader 2].

The relationships developed between the initiative, the medical centre, relevant NHS departments, and the leisure centre were perceived as important in integrating the service into a more complete health care system: *“The health professionals … become very supportive … To an extent, they changed the way they operate to consider Active Life … They see Active Life as a logical extension of what they are doing”* [Project Leader 1].

**Outcomes:** Active Life developed relations with, and between, different community actors, facilitating strong ties between the medical centre and smaller non-profit organisations, acting as a boundary spanner. As such, cross-collaboration, with strong involvement and consideration of community voices was essential in the design and implementation of new services: *“Involving all the other third sector organisations … ‘let's go all together and grow this vision of a healthy town’ … If the community stays in charge, and the community drives rather than the NHS, I think we would achieve that much, much better … [and have a] better chance to achieve something like that”* [Project Leader 1].

## 5 Discussion

**Fig. 2:**
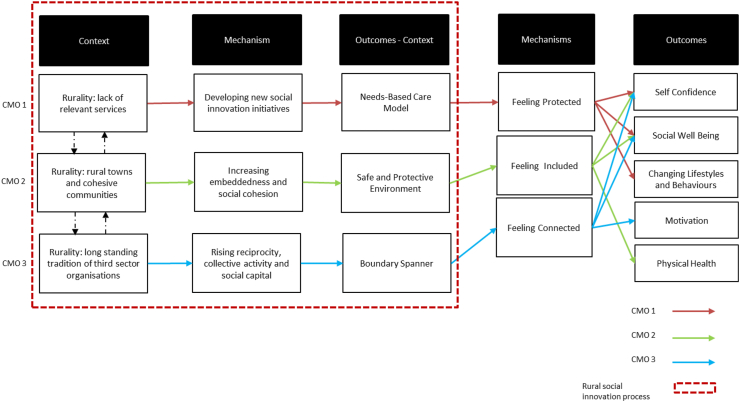
Process of Rural Social Innovation – context, mechanisms and outcomes.

The study aimed to explore how *rurality* might affect the social innovation process. Building upon the social innovation analytical framework that explored ‘what works’ and ‘for whom’ components of realist evaluation theory (Calò et al., 2019), we focus on the third component of the theory - ‘circumstances’ - and further develop Calò et al.’s (2019) framework by unpacking the context and mechanisms of *rural* social innovation. Fig. 2 summarises three CMO statements connecting them to the findings of the rural social innovation intervention in terms of context, mechanisms and outcomes.

Our empirical data shows how rurality influences the implementation of social innovation. First (CMO1), we observe that although negative in itself, a lack of relevant services (Farmer and Nimegeer, 2014) can trigger rural communities to develop new services and address local challenges through a *reactive* social innovation process. This might commonly be associated with roll-back neoliberalisation, but there is no evidence in this case that a reduction in state provision was facilitated by social innovation mitigating the impacts. In our study, the social innovation initiative was also *proactive* as it engaged the local community, health professionals and community organisations to better understand people's needs and explore potential solutions through a ‘fit for purpose’ intervention – a service perceived as appropriate in the local setting. The development of the ‘needs-based care model’ was ‘the ideal solution’ for (and run by) the community as it identified local needs as well as the local support needed to implement an innovative solution. The ‘needs-based care model’ then triggers feelings of protection among beneficiaries, helps to overcome a fear of undertaking physical activity, increases self-confidence, improves social well-being and supports lifestyle change.

Second (CMO2), the rural location where the initiative took place was identified by stakeholders as a cohesive town in which people know and trust each other and where community members work together to enhance the quality of life of local residents. This context helped to further develop a sense of community, strong mutual knowledge and embeddedness *inside the initiative*. New relationships and strong social ties between staff, beneficiaries and other participating stakeholders led to the development of a safe and protective environment, which reinforced a sense of belonging and inclusiveness within the community and led to improved physical health, confidence and social well-being.

Third (CMO3), the rural town under study was characterised by a long-standing presence of third sector organisations as well as community and social enterprises. Professionals supporting the social innovation initiative were also involved in setting up, developing and running other local initiatives. These features are recognised in the rural literature as helpful in supporting social entrepreneurship (Munoz et al., 2015; Williams, 2007). Here, we also see the applicability of these rural features in supporting the rural social innovation process. This specific characteristic was also important in boosting reciprocity, collective activity and social capital *outside the initiative*. Local social networks led the social innovation initiative to be a ‘boundary spanner’, creating relationships within and between different community actors. This mechanism helped to reach beneficiaries with different chronic conditions, boosting their motivation and confidence in pursuing physical activity and getting back to a day-to-day routine.

Considering our three CMOs, the success of the rural social innovation process depends on a sufficient level of active civic participation and self-help (Farmer et al., 2008). This suggests that community readiness is essential and rural communities characterised by a lack of engagement or capacity to develop and run an initiative might be deprived of the opportunity to introduce social innovation. Rural embeddedness and social cohesion – common features of many rural communities (Granovetter, 1985; Jack and Anderson, 2002) – are also crucial in strengthening rural social innovation. Rural residents participating in a social innovation initiative interact and support each other, developing and enhancing social bonds. Indeed, the geographical context of rural towns and villages can facilitate this process as residents of small communities perceive each other as neighbours – something that might be lost or not present in a large urban centre. Finally, we see that close-knit ties evident in rural communities as well as strong social capital and networking facilitate the rural social innovation process (Richter, 2019), resulting in relevant stakeholders being willing to find time to collaborate. Again, this characteristic might be specific to rural professionals who understand the need to work together to address local challenges. Working in ‘silos’ might simply not work in rural locations and breaking those silos is facilitated by long-lasting familiarity and personal as well as professional relationships between local actors. This, in time, enables rural social innovation to thrive.

Such assets may, in principle, be diluted by roll-back and roll-out neoliberalisation but in our study there was no evidence of such an effect. While the lack of services which stimulated social innovation in CMO1 might well have been associated with roll-back neoliberalisation, the process of innovation was proactive as much as reactive, leading to the development of a new ‘needs-based care model’. In all three CMOs the initiatives were not simply ‘self-help’ but involved a range of partners and assets while under the initiative and control of people ‘in place’.

Interestingly, outcomes of the social innovation process might not be specific to rural areas, nor the same in all rural areas. Instead, the way to get to the desired outcomes is conditioned by rural and other factors shaping contexts and mechanisms of rural social innovation. As such, rural context and associated rural mechanisms do matter. It is important to emphasise, however, that different rural localities might have different resources to address local challenges and that, apart from recognising this diversity, capacity-building may be necessary to enable social innovation. As such, the social innovation process varies from one case to another, although the challenge being addressed might be similar. Indeed, this flexibility, adjustment and ‘re-contextualisation’ of the rural social innovation process (Richter, 2019) has consequences for rural social innovation policy and practice, which should not be ‘over prescribed’ or standardised – instead, it must consider both the visible (e.g. lack of local services) and invisible (e.g. reciprocity, social capital and connectedness) characteristics of rurality.

## 6 Conclusions

The originality of this paper derives from using realist evaluation theory to develop our understanding of how rurality affects the social innovation process and its outcomes. To our knowledge, the use of realist evaluation in this context is novel, yet it is helpful in evidencing pathways of social innovation in rural locations. As such, we add to the existing literature in this field and address existing knowledge gaps by identifying Context-Mechanism-Outcome configurations of rural social innovation.

Our paper shows that specific characteristics of rural places can act as stimuli to social innovation, fostering regional development (Castro-Arce and Vanclay, 2020; Christmann, 2014) and helping to address some of the socio-economic challenges and geographical disadvantages faced by rural residents (Anderson and Lent, 2019; Best and Myers, 2019; Jack and Anderson, 2002, Steiner et al., 2019). Interestingly, the positive outcomes of a social innovation intervention can potentially be rooted in rural peculiarity and problematic rural contexts. For instance, withdrawal of services from rural areas (often associated with roll-back neoleralisation) can act as a stimulus of social innovation. Push factors, born out of necessity, lead to *reactive* social innovation and pull factors, derived through harnessing perceived opportunities in the environment, lead to *proactive* social innovation. Importantly, push factors do not undermine the establishment of social innovation – indeed, they can actually promote social innovation by strengthening its validity. For this to happen, however, there needs to be a sufficient level of buy-in from local communities. Indeed, without embeddedness, trust and social capital, the rural social innovation process might not be successful. Indeed, ‘although rural communities do not control all the conditions that affect them, they have the ability to adapt to some structural features’ (Steinerowski and Steinerowska-Streb, 2012, p. 167) and identify tailored rural solutions to specific rural problems. Context, therefore, can create both challenges and solutions, and influence the type and form of mechanisms used to achieve a desirable social innovation outcome.

As evidenced in other studies (Neumeier, 2017), a collaborative geographical space is essential for social innovation. In our case, rurality and the characteristics of rural communities – strong social capital and a willingness to address local issues – became a collaborative platform for rural social innovation. Importantly, however, in addition to place and context time is also relevant for inducing social innovation. For instance, the social innovation initiative described in this paper took place in times of public service withdrawal and limited and shrinking rural service delivery, and at a moment when social innovation and the notion of empowered communities ‘doing things for themselves’ is being promoted in policy documents (Markantoni et al., 2018), in accordance with narratives of neoliberalisation. Hence, place and time are important drivers and influence how a social innovation initiative is initiated and implemented. Place and time might also determine whether the social innovation is successful in achieving its outcomes.

Although we develop new knowledge, we recognise some limitations of our study and resultant future research avenues. Firstly, we have explored one social innovation initiative and one rural location and, as such, our findings should be verified, or challenged, in future rural studies. Secondly, exploring a similar initiative in an urban area could build an understanding of what would happen to the social innovation without the rural contextual characteristics. An urban-rural comparative study would also help to understand which of the two contexts provides more fertile ground for successful social innovation processes.

## Footnotes

1 Detailed results of the first two cycles of analysis are reported in Calò et al. (2019).
